# Two-stage approach to obturator hip dislocation with subcapital femoral neck fracture: a case report

**DOI:** 10.11604/pamj.2025.52.157.50375

**Published:** 2025-12-14

**Authors:** César Gonzalez-Cancino, José Barraza-Franco

**Affiliations:** 1 Department of Orthopaedic and Traumatology Surgery, Hospital San Lucas, Carretera Tuxtla Villaflores 3839, Tuxtla Gutíerrez, Chiapas, Mexico,; 2 Angeles Hospital Puebla, Puebla, Mexico

**Keywords:** Anterior hip dislocation, femoral neck fracture, intrapelvic femoral head, stoppa approach, case report

## Abstract

Anterior dislocation of the hip associated with an ipsilateral femoral neck fracture is exceedingly rare and typically results from high-energy trauma. We describe the case of a 33-year-old man who was engaged in a car crash and suffered numerous injuries, among them an anterior hip dislocation with the femoral neck trapped in the obturator foramen. The patient was treated in a single surgical procedure using a dual approach: resection of the femoral head through the Stoppa approach and placement of a primary total hip replacement through the Hardinge approach. The procedure was completed without complications, and rehabilitation included early mobilization with full weight-bearing at two weeks. At 24 months, the patient demonstrated preserved muscle strength, stable radiographs without loosening, and a Harris Hip Score of 86. This case highlights a rare injury pattern and supports the use of a dual-approach single-stage procedure as a safe and effective treatment strategy.

## Introduction

Anterior hip dislocation is uncommon, accounting for 7-13% of all hip dislocations [1], while femoral neck fractures comprise 3-5% of fractures overall [2]. The coexistence of both is exceptional, typically following high-energy trauma, since the surrounding musculature resists such displacement. Given the paucity of reported cases, evidence-based guidelines for management remain elusive, and long-term prognosis is poorly defined.

## Patient and observation

**Patient information:** a 33-year-old male with no significant past medical history sustained a high-velocity rollover motor vehicle collision with ejection.

**Timeline of current episode:** for the accident, he suffered a traumatic brain injury followed by transient loss of consciousness. He required a 10-day intensive care admission for moderate head trauma. Upon stabilization, orthopedic evaluation was carried out.

**Clinical findings:** the orthopedic findings demonstrated a shortened left lower limb (≈4 cm), fixed external rotation (75°), severe pain with attempted movement, and periarticular swelling in the hip area. Concomitant injuries included right shoulder dislocation, right radioulnar fracture, right clavicular fracture, and left zygomatic arch fracture.

**Figure 1:**
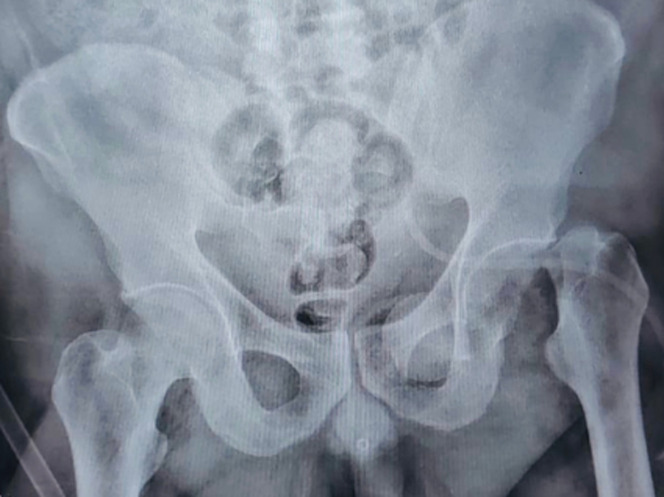
anteroposterior radiograph of the pelvis showing anterior hip dislocation and a femoral head fracture

**Figure 2:**
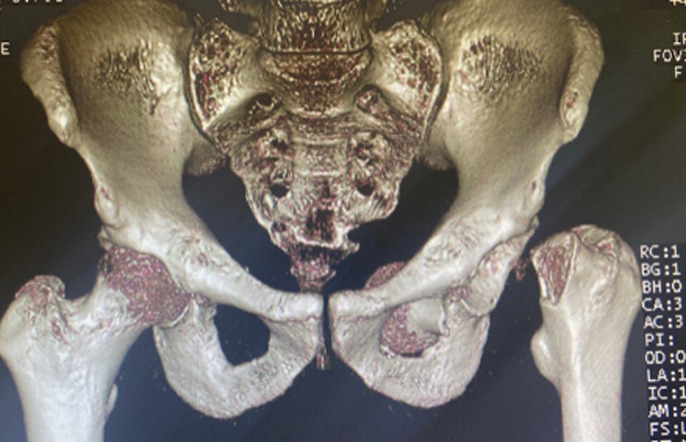
three-dimensional computed tomography image showing the femoral head in an intrapelvic position

**Diagnostic assessment:** plain radiographs revealed anterior dislocation of the left hip, accompanied by a femoral neck fracture and migration of the femoral head into the obturator foramen within the pelvis (Figure 1). Computed tomography corroborated the intrapelvic location (Figure 2).

**Figure 3:**
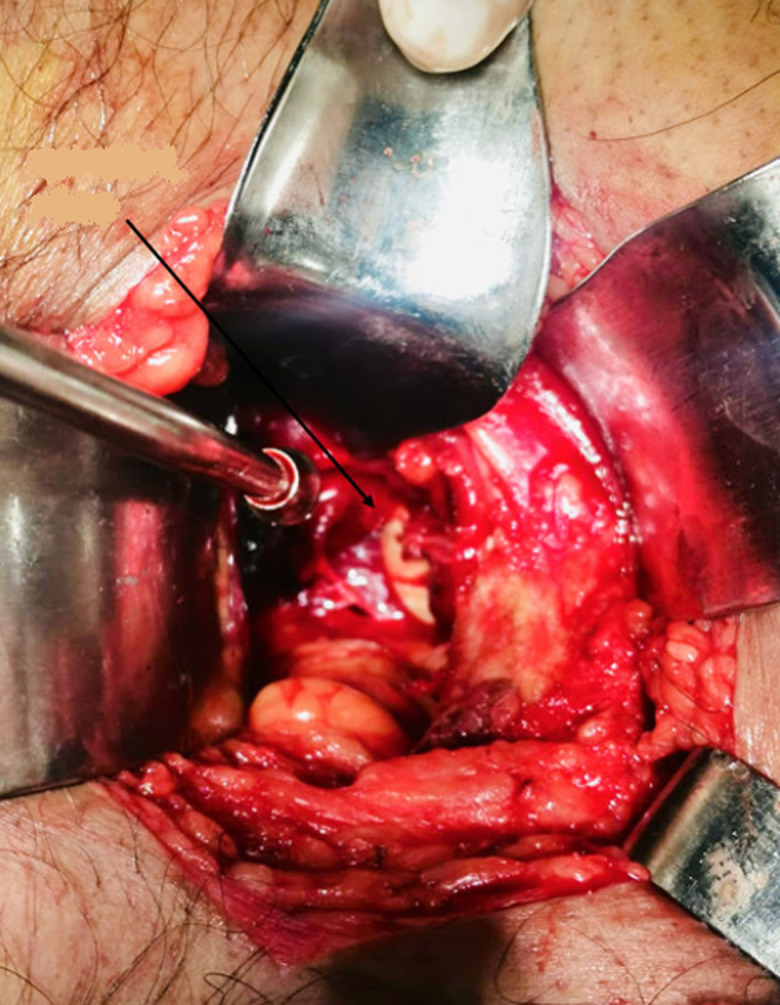
intraoperative view of the femoral head in its intrapelvic position through the stoppa approach

**Figure 4:**
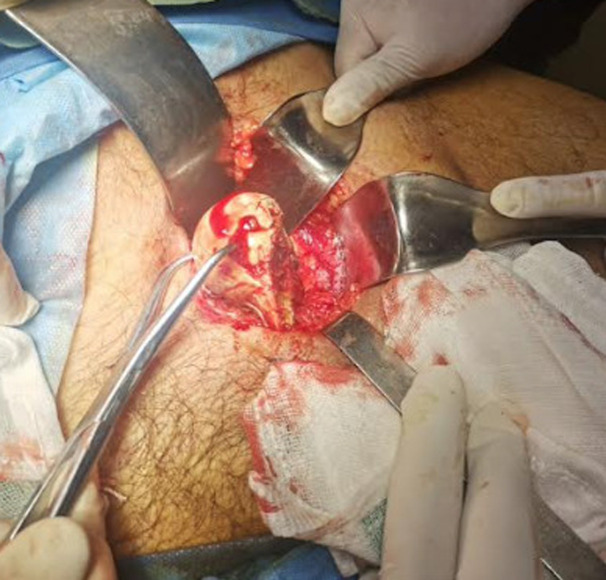
femoral head removed using a clamp

**Figure 5:**
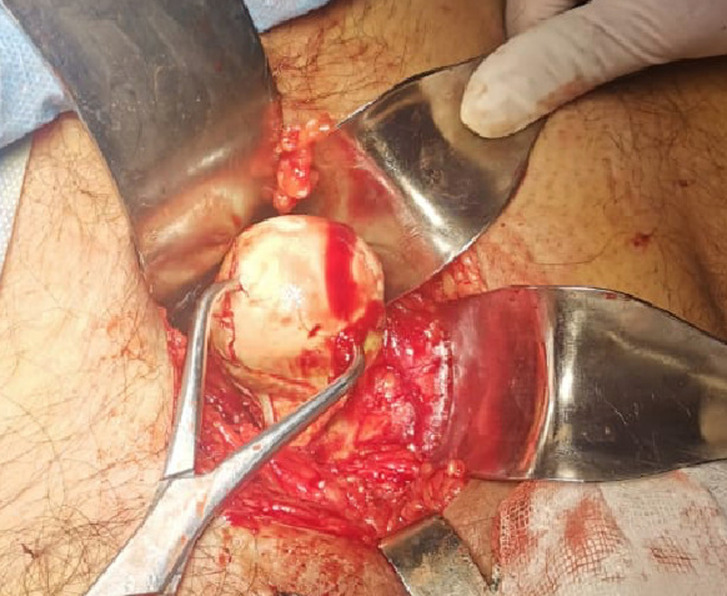
cartilage damage on the anterosuperior surface of the femoral head

**Figure 6:**
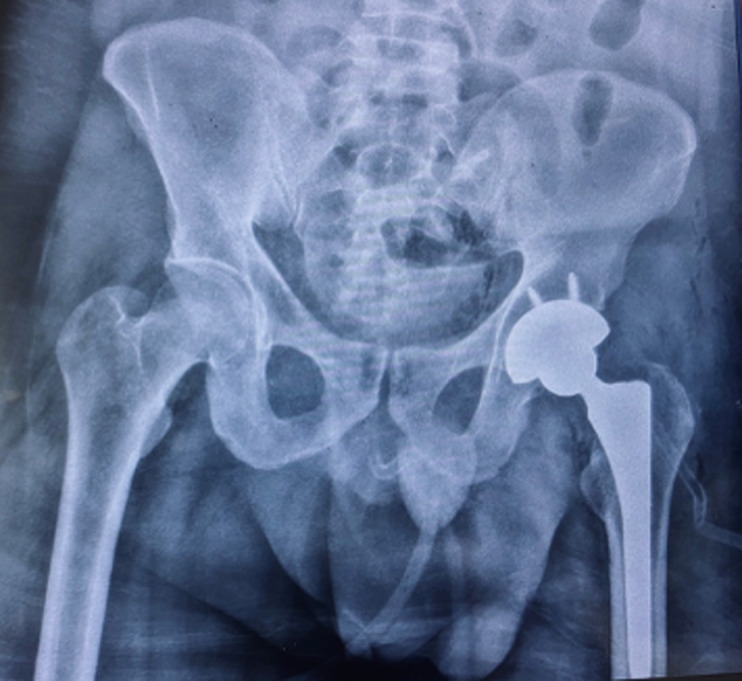
immediate postoperative anteroposterior radiograph of the pelvis after total hip arthroplasty

**Therapeutic interventions:** given polytrauma, definitive hip surgery was deferred for 26 days. A dual-stage, single-anesthetic approach was selected. Initially, with the patient supine, a Stoppa-type intrapelvic approach was performed. Intrapelvic exploration identified the femoral head penetrating the obturator membrane and lodged within the foramen (Figure 3). It was mobilized using blunt dissection and clamp forceps and removed without complications (Figure 4). The femoral head showed marked anterosuperior cartilage damage (Figure 5). Following wound closure, the patient was arranged in a side-lying position. A direct lateral (Hardinge) approach was undertaken, involving gluteal tenotomy, capsulectomy and excision of fibrotic tissue. An uncemented total hip prosthesis (B. Braun) was implanted without difficulty. Estimated blood loss was 200 mL, and no complications occurred (Figure 6).

**Figure 7:**
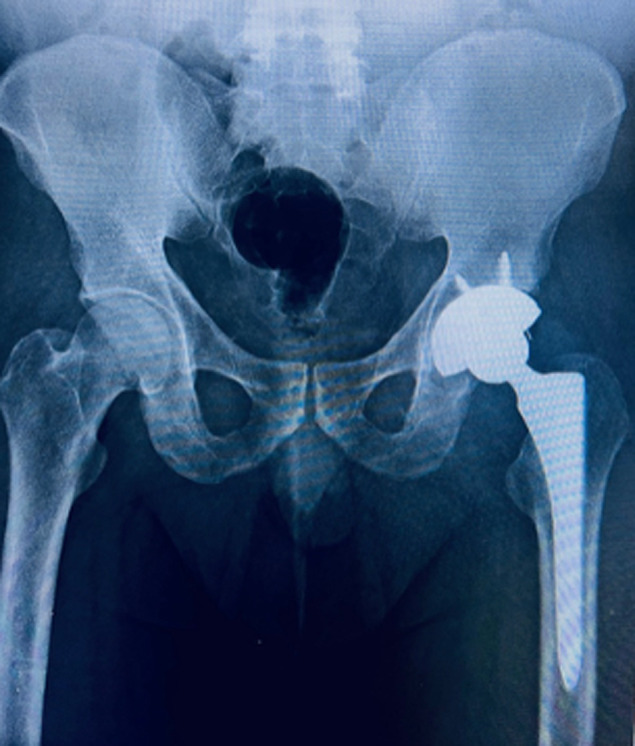
anteroposterior radiograph of the pelvis at 2-year follow-up after total hip arthroplasty

**Follow-up and outcome of interventions:** postoperatively, early passive and active hip mobilization was initiated. Full weight-bearing ambulation began two weeks post-surgery. At 24-month follow-up, the patient reported only minimal pain during hip flexion, no claudication, and had preserved muscular strength (Daniel´s scale 5/5). Radiographs revealed no evidence of lysis or aseptic loosening of the prosthesis (Figure 7). The Harris Hip Score was 86 points, indicating a favorable outcome.

**Patient perspective:** the patient was satisfied with the medical care he received, the surgical procedures performed, and his recovery.

**Informed consent:** written informed consent was obtained from the patient for participation in this publication.

## Discussion

Simultaneous anterior hip dislocation with ipsilateral femoral neck fracture is exceedingly rare, and is associated with high-energy mechanisms in younger individuals; nearly 75% cases occurring before the age of 50 [3]. The sequelae are usually severe, with avascular necrosis of the femoral head being the most significant complication, and subsequent hip joint osteoarthritis. Bassett *et al*. [4], detailed that forced abduction with external rotation drives the greater trochanter and femoral neck against the rim of the acetabulum, levering the femoral head anteriorly. A flexed hip predisposes to obturator dislocation, while extension favors public dislocation. Liu *et al*. [5] presented a two-impact model in which the proximal femoral shaft strikes the outer and superior sections of the femoral head, propelling it anteroinferiorly through the sartorius pathway into the obturator area, where it becomes incarcerated beneath the inferior pubic branch and entrapped within the obturator foramen. Reported treatment strategies for this condition range from conservative management and internal fixation (cannulated screws or dynamic hip screw), partial hip replacement and total hip arthroplasty (THA). Management is challenging and must be individualized, as the goal is to provide an effective treatment with good medium- and long-term outcomes. Hip dislocation may also cause traction, compression, or spasm of the femoral, deep femoral, and circumflex arteries [6].

These vascular changes can sometimes be reversed by early reduction of the dislocation [7]. Preservation of the medial circumflex femoral artery and the retinacular vessels is an important factor when considering femoral head-sparing procedures [8]. Jain *et al*. [9], described a situation handled with uncemented total hip arthroplasty for a hip dislocation accompanied by a fracture in the femoral neck on the same side, resulting in satisfactory hip movement after six weeks. They determined that the selection of surgical intervention ought to take into account the timing of the injury, the age of the patient, individual preferences, and the extent of cartilage damage in the femoral head. In our case, we chose THA because 26 days had elapsed since the injury, and we considered the femoral head circulation irreversibly compromised. It has been noted that in two instances of internal hip dislocation of the obturator type, the posterolateral method was ineffective in extracting the dislocated femoral head from the obturator [10]. Using traditional approaches for THA to remove the femoral head directly from the obturator also makes hemostasis difficult if bleeding occurs. Due to the complex vascular anatomy of the obturator region coupled with the amount of fibrosis present due to the time of the lesion, we opted for a Stoppa-type intrapelvic approach, which allowed direct visualization and removal of the femoral head safely and efficiently, minimizing additional trauma and reducing the risk of bleeding compared with attempting removal via the direct lateral (Hardinge) approach.

## Conclusion

This report outlines an uncommon instance of dislocation of the anterior hip with concomitant intrapelvic femoral neck fracture. A single-stage dual approach proved safe and effective. This allowed direct removal of the femoral head while mitigating neurovascular complications and facilitated implantation of total hip prosthesis via conventional approach without additional tissue manipulation or damage. Such a technique may represent a suitable management strategy in similar traumatic scenarios.
